# Monitoring Notch Signaling-Associated Activation of Stem Cell Niches within Injured Dental Pulp

**DOI:** 10.3389/fphys.2017.00372

**Published:** 2017-05-30

**Authors:** Thimios A. Mitsiadis, Javier Catón, Pierfrancesco Pagella, Giovanna Orsini, Lucia Jimenez-Rojo

**Affiliations:** ^1^Orofacial Development and Regeneration, Faculty of Medicine, Institute of Oral Biology, ZZM, University of ZurichZurich, Switzerland; ^2^Department of Medical Basic Sciences, Faculty of Medicine, University CEU-San PabloMadrid, Spain; ^3^Department of Clinical Sciences and Stomatology, Polytechnic University of MarcheAncona, Italy

**Keywords:** stem cells, dental pulp, niches, tooth injury, regeneration, cell proliferation, Notch signaling, Notch2

## Abstract

Dental pulp stem/progenitor cells guarantee tooth homeostasis, repair and regeneration throughout life. The decision between renewal and differentiation of these cells is influenced by physical and molecular interactions with stromal cells and extracellular matrix molecules forming the specialized microenvironment of dental pulp stem cell niches. Here we study the activation of putative pulp niches after tooth injury through the upregulation of Notch signaling pathway. Notch1, Notch2, and Notch3 molecules were used as markers of dental pulp stem/progenitor cells. Upon dental injury, Notch1 and Notch3 are detected in cells related to vascular structures suggesting a role of these proteins in the activation of specific pulpal perivascular niches. In contrast, a population of Notch2-positive cells that are actively proliferative is observed in the apical part of the pulp. Kinetics of these cells is followed up with a lipophilic DiI labeling, showing that apical pulp cells migrate toward the injury site where dynamic regenerative/repair events occur. The knowledge of the activation and regulation of dental pulp stem/progenitor cells within their niches in pathologic conditions may be helpful for the realization of innovative dental treatments in the near future.

## Introduction

Biological repair and regeneration is an attractive alternative and/or complement to prosthetic replacement of tissues and organs. Cell-based therapeutic approaches consist of *in vitro* manipulation of stem cells and their consequent administration to patients (Passier et al., 2008; Segers and Lee, 2008; Djouad et al., 2009; Robinton and Daley, 2012; Shevde, 2012; Bender, 2016). Stem cells are defined by their dual capacity of self-renewal and multipotency (referred to as stemness) (Thomson et al., 1998; Shevde, 2012). These properties make stem cells extremely interesting for clinical tissue engineering applications (Bianco and Robey, 2001; Mitsiadis et al., 2012; Aurrekoetxea et al., 2015; Mele et al., 2016). Stem cells have been identified in the pulp of deciduous and adult permanent teeth (Mitsiadis et al., 2015; Miran et al., 2016). These cells are able to differentiate both *in vivo* and *in vitro* into many cell types such as odontoblasts, osteoblasts, chondrocytes, adipocytes, and neuronal cells (Bluteau et al., 2008; Mitsiadis et al., 2015). There is increasing evidence for the existence of more than one stem/progenitor cell populations within the dental pulp (Mitsiadis et al., 2011; Ducret et al., 2016).

Dental injuries are often lethal for the odontoblasts at the proximity of the lesion site, an event that triggers activation of dental pulp stem/progenitor cells. These cells proliferate, migrate, and finally differentiate into odontoblast-like cells that elaborate the reparative dentin (Mitsiadis and Rahiotis, 2004). However, the nature and exact location of these mesenchymal cell populations are not yet known. Niches consist of specific and protected anatomic locations housing stem/progenitor cells and enabling them to self-renew. Stromal cells belonging to a niche control stem cell behavior through cell-cell interactions, soluble factors, and specialized extracellular matrices (Scadden, 2006; Djouad et al., 2009; Shaker and Rubin, 2010; Oh and Nör, 2015; Pagella et al., 2015). This particular microenvironment permits stem/progenitor cells to survive, to change their number and fate, regulating thus their participation in tissue maintenance, repair and/or regeneration. Therefore, it is essential to identify stem cell niches within the dental pulp in order to understand the mechanisms and the microenvironment that support the survival of stem/progenitor cells in teeth.

Notch molecules are important regulators of the stem cell fate, with capacity to induce cell proliferation and/or differentiation (Hori et al., 2013). The close association of dental pulp mesenchymal cells and neo-vessels in dental diseases (e.g., carious lesions, injuries) and their relation to Notch signaling pathway may be critical in the regulation of stem cells to differentiate into odontoblast-like cells (Lovschall et al., 2007; Mitsiadis et al., 2011; Oh and Nör, 2015). Notch proteins form a family of evolutionary conserved transmembrane receptors that determine cell fate (Artavanis-Tsakonas and Muskavitch, 2010). In mammals, the four Notch receptors (i.e., Notch1, Notch2, Notch3, Notch4) are activated following direct contact with their five ligands: Jagged1 (Jag1), Jag2, Delta-like1 (Dll1), Dll3, Dll4. Upon ligand-receptor binding, the Notch protein is cleaved and its intracellular domain (NICD) translocates to the nucleus, where it associates with the DNA binding protein RBP-Jk to activate transcription (Artavanis-Tsakonas and Muskavitch, 2010; Hori et al., 2013). It has been reported that Notch activation promotes stem cell maintenance (Androutsellis-Theotokis et al., 2006; Artavanis-Tsakonas and Muskavitch, 2010).

Although Notch signaling has been exhaustively studied during tooth development, pathology and repair, its role in regulating the behavior of dental pulp stem/progenitor cells after injury remains elusive (Mitsiadis et al., 1999, 2003; Sun et al., 2014). To address such questions, experiments using loss- and/or gain-of-function transgenic animal models are necessary. In the present manuscript, as a first attempt to investigate this issue, we studied the correlation between the expression of Notch receptors and migration of apical pulp cells upon injury at the tooth crown.

## Materials and methods

### Tissue preparation and dental explant cultures

All mice (C57Bl/6; postnatal day 6–8) were maintained and handled according to the Swiss Animal Welfare Law and in compliance with the regulations of the Cantonal Veterinary office, Zurich (License 11/2014). The licensing committee of the Gesundheltsdirektion Kanton Zürich approved all experimental protocols (Versuch Nr. 11/2014 “Study of the function and potency of human and mouse dental stem cells after *in vitro* culture”). Mice were sacrificed by cervical dislocation and their mandibles were dissected out and cultured *in vitro*. Cavity preparations were performed in 20 first mandibular mouse molars using a 20-gauge drill, while 4 intact molars were used as a control. Four hours after the operation, the first molars were carefully extracted from the mandibles. Mandibles and dental explants were cultured in a medium consisted of Dulbecco's Modified Eagle Medium (DMEM; GibcoBRL), 20% fetal calf serum (FCS; GibcoBRL), 20 units/ml penicillin/streptomycin (GibcoBRL), and glutamine at 37°C/5% CO_2_. Most of the molars were used for DiI labeling experiments, cell proliferation essays, and whole mount immunohistochemistry, while other molars were sectioned with a cryostat (12–14 μm sections) and used for immunohistochemistry and cell proliferation essays. Molars from more aged mice could not be used, as it was impossible to remove the hard mineralized tissues (i.e., dentin, enamel) without causing sparse damage to the pulp tissue. Cultures were maintained for at most 4 days, since pulps cultured for longer periods showed clear signs of pulp necrosis.

### Immunohistochemistry and immunofluorescence

Polyclonal antibodies against the mouse Notch1, Notch2, and Notch3 proteins were used (Mitsiadis et al., 1995, 1999, 2003). Immunohistochemistry on sections were performed as described previously (Mitsiadis et al., 1995, 1999, 2003). Briefly, the antibodies were applied for 2 h at 37°C, thereafter the sections were incubated with the biotinylated secondary antibody for 1 h and finally with peroxidase-conjugated streptavidine for 10 min. Peroxidase was revealed with 3-amino-9-ethylcarbazole (AEC) reaction solution. In control sections the primary antibodies were omitted.

For immunofluorescence on the entire dental pulp, tissues were first fixed in 1% paraformaldehyde (PFA) at 4°C overnight and then washed with phosphate-buffered saline (PBS)/0.2% bovine serum albumin (BSA)/0.3% Triton X-100 overnight. Samples were then incubated with Notch2 primary antibody during 5 days at 4°C. Thereafter, tissues were washed with PBS and post-fixed 30 min at RT in 4%PFA and incubated in HCl (2 M) for 30 min at 37°C prior to the incubation with the anti-BrdU antibody for 7 days at 4°C. Secondary antibodies were added separately and each of them incubated during 3 days at 4°C. Samples were then mounted with 2.5% 1,4-diazobicyclo-[2.2.2]-octane (DABCO, Sigma, D2522) mounting medium and analyzed using a Leica SP5 confocal microscope.

### Cell proliferation analysis

Cell proliferation in cultured dental explants was analyzed by using a bromodeoxyuridine (BrdU) cell proliferation kit (Boehringer Mannheim). The explants were cultured for an additional time (i.e., 2–4 h) with BrdU, according to the manufacturer's instructions. BrdU-positive cells were monitored after staining with an anti-BrdU antibody and detected by means of a Vectastain ABC kit (Vector). Whole mount immunohistochemistry was performed as earlier described (Mitsiadis et al., 1995).

### DiI labeling and fate mapping of dental cells

Immediately after extraction, cells located at the apical part of dental pulp of both intact and injured first molars were labeled with DiI (Molecular probes cell tracker CM-DiI, C-7000). DiI is a lipophilic dye that intercalates in the cell membrane marking small groups of cells. DiI was prepared in ethanol (EtOH) at 2.5 μg/μl. This stock solution was then diluted 1–9 in 0.2 M sucrose and warmed. DiI was injected by a mouth-controlled borosilicate glass micropipette. After DiI labeling, dental explants were cultured for 4 days. Thereafter, explants were fixed with 4%PFA in Dulbecco's PBS at 4°C for 2 h. After fixation, the mineralized part of the majority of the teeth was carefully removed in order to obtain dental pulp tissues free of dentin and enamel. The fate of the labeled cells was assessed in transmitted light and fluorescence images, which were captured with a Zeiss Axioscope equipped with a CCD camera. The transmitted light and fluorescence images were finally merged.

## Results

### Distribution of Notch proteins in injured teeth

**Figure 1:**
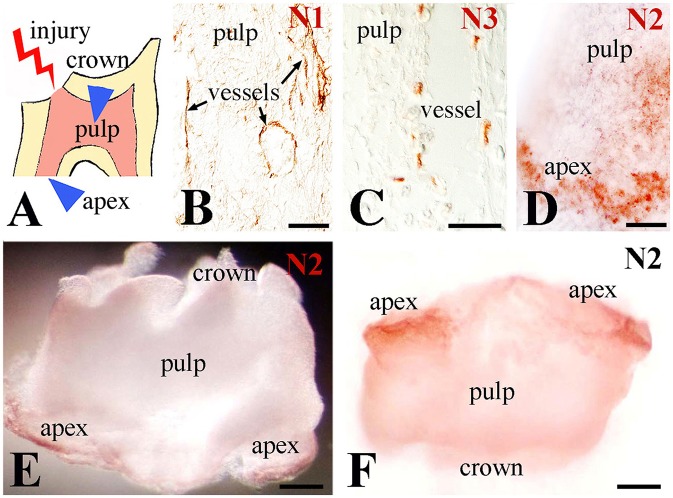
Distribution of Notch proteins in the injured dental pulp. **(A)** Schematic illustration showing dental pulp injury after cavity preparation. Arrowheads indicate the areas of Notch activation within the pulp. **(B)** Notch1 (N1) staining is visible in cells lining blood vessels within the central part of the pulp. **(C)** Notch3 (N3) labeling is detected in perivascular cells of the central part of the pulp. **(D)** Notch2 (N2) staining is mainly found in apical pulp cells at the tooth apex. **(E,F)** Whole mount immunostaining showing Notch2 protein distribution at the apical part of an injured pulp. Scale bars: 50 μm **(B–D)**, 200 μm **(E,F)**.

Upon injury of developing first molars (Figure 1A), immunohistochemistry on sections showed that Notch1 and Notch3 expression in the central part of the dental pulp is confined to cells forming vascular structures (Figures 1B,C). In contrast, Notch2 staining is detected in cells of the apical pulp (Figure 1D). This was confirmed by whole mount immunohistochemistry, clearly showing Notch2-positive cells at the apical part of the pulp (Figures 1E,F). Immunostaining against the three Notch proteins was not detected in the pulp of intact teeth (data not shown) that is in accordance with our previous results (Mitsiadis et al., 1999, 2003; Sun et al., 2014).

### Correlation of Notch2 protein distribution and cell proliferation at the apical part of the injured pulp

**Figure 2:**
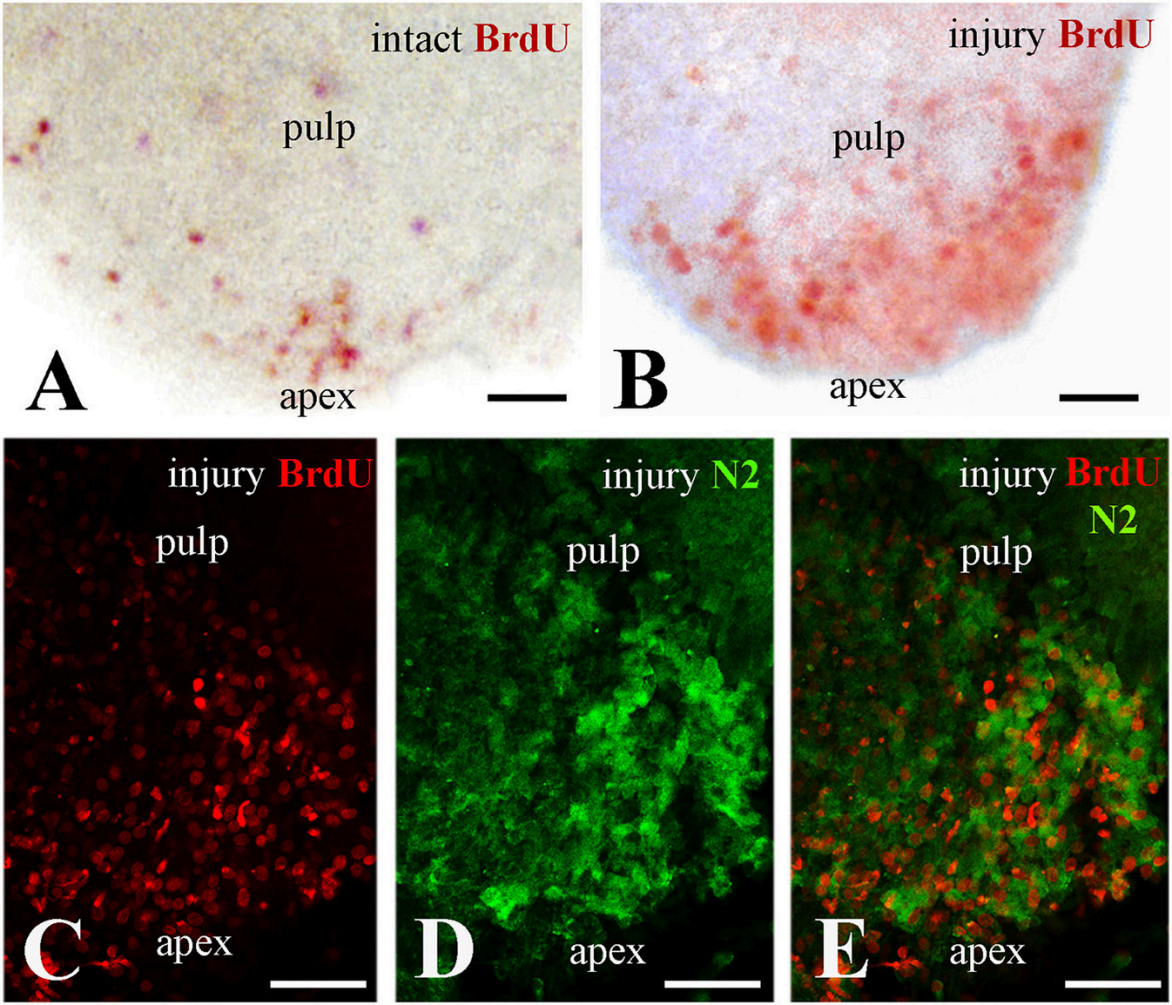
Proliferation of dental pulp cells correlates with Notch2 protein expression in the apex of injured molar teeth. BrdU in red color, Notch2 (N2) staining in green color. **(A)** Proliferating dental pulp cells in intact teeth. **(B)** Proliferating pulp cells in injured teeth. Note that cell proliferation increases upon injury. **(C–E)** Confocal microscope analysis showing co-expression of BrdU **(C)** and Notch2 **(D)** in the apical part of the pulp after merging of the pictures **(E)**. Scale bars: 50 μm **(A–E)**.

In an attempt to ascertain whether Notch2 protein expression in the apical pulp was correlated with cell proliferation, healthy and injured dental pulp explants were cultured in presence of BrdU for up to 4 h before fixation. BrdU whole mount immunostaining revealed that while in the intact teeth only few cells proliferate at the apical part of the pulp (Figure 2A), cell divisions significantly enhance in this area upon tooth crown injury (Figure 2B). Double immunofluorescence on sections of injured pulp explants, analyzed in confocal microscopy, revealed that proliferating cells at the apical pulp part also express the Notch2 protein (Figures 2C–E).

### Lineage tracing of apical dental pulp cells in intact and injured teeth

**Figure 3:**
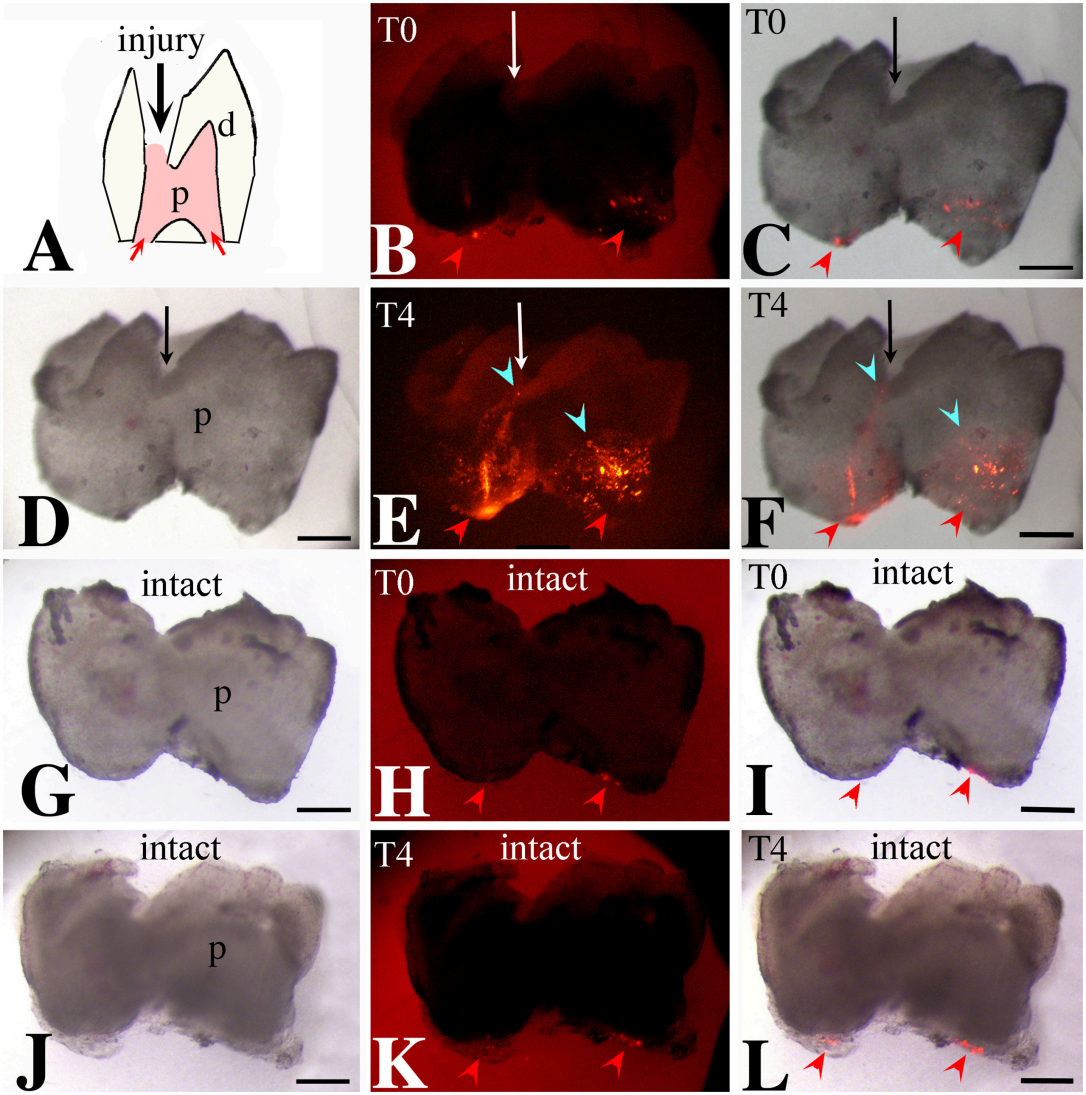
Migration of DiI-labeled cells from the apical part of the pulp toward the injury site. Bright-field pictures **(D,G,J)**, dark-field pictures **(B,E,H,K)**, merged pictures **(C,F,I,L)**. Red arrows and arrowheads indicate the areas of DiI (red color) injection. Light blue arrowheads indicate the final location of migrating DiI-positive cells. Black and white arrows indicate injured teeth. **(A)** Schematic illustration showing dental pulp injury after cavity preparation. **(B,C)** Dental pulps immediately after injury and DiI injection (T0). **(D–F)** Dental pulps 4 days after cavity preparation and DiI injection (T4). **(G–I)** Intact dental pulps immediately after DiI injection (T0). **(J–L)** Intact dental pulps 4 days after DiI injection. Abbreviations: d, dentin; p, pulp; T0, time zero days; T4, time 4 days. Scale bars: 200 μm **(B–L)**.

**Figure 4:**
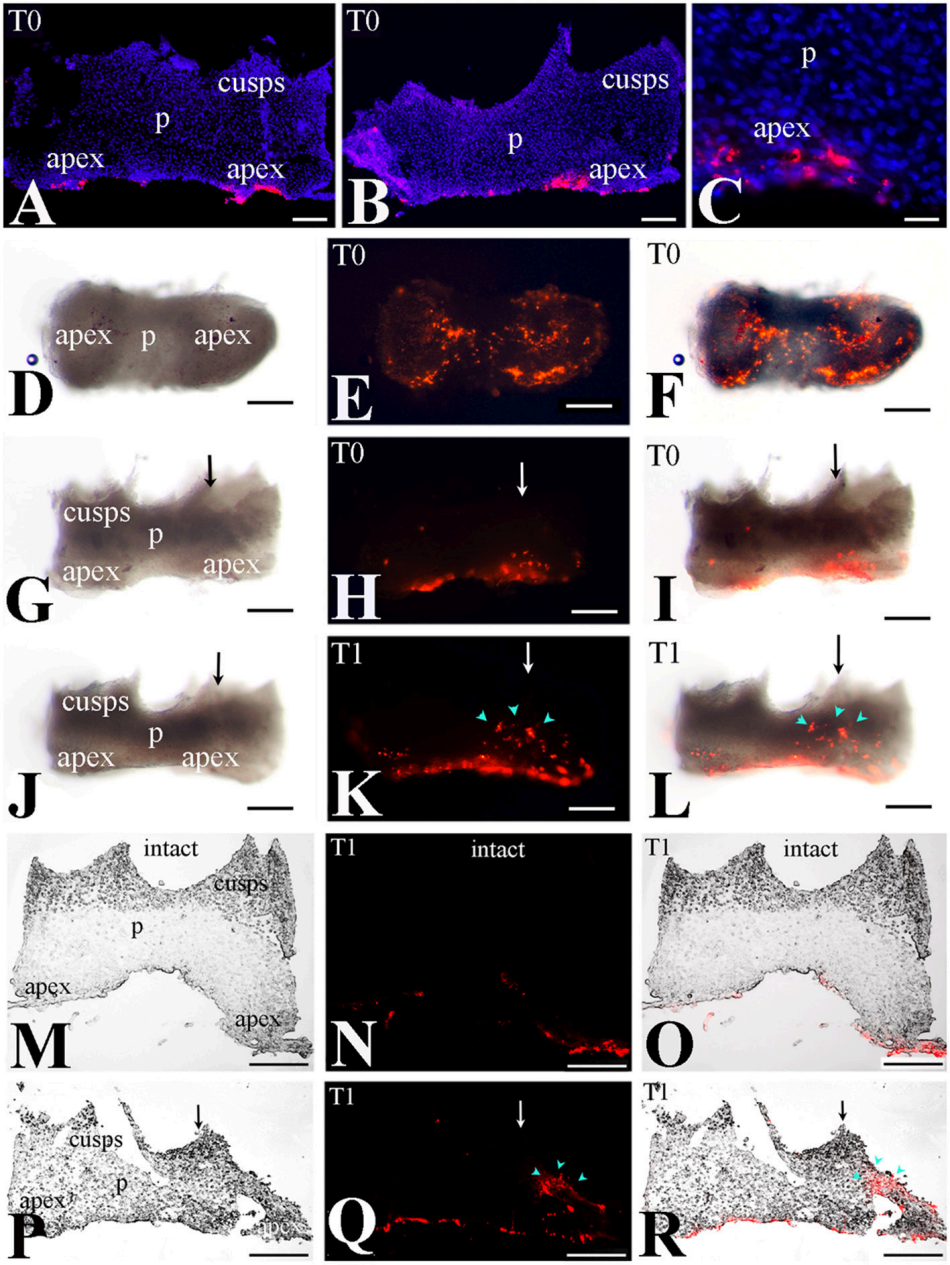
Migration of DiI-labeled cells from the apical part of the pulp toward the injury site. **(A–C)** DiI labeled cells at the apical part of the pulp (T0). Dark-field pictures. DiI labels cells in red color while DAPI stains in blue color the nuclei of pulp cells in cryosections. **(D–L)** Migration of DiI-labeled cells from the root apex toward the injury site - whole pulp view. Bright-field pictures **(D,G,J)**, dark-field pictures **(E,H,K)**, merged pictures **(F,I,L)**. **(D–I)** Dental pulps immediately after injury and DiI injection (T0). **(J–L)** Dental pulps 1 day after cavity preparation and DiI injection (T1). Black and white arrows indicate the area of the tooth injury. DiI-positive pulp cells are seen in red color. Light blue arrowheads indicate the final location of migrating. **(M–R)** Migration of DiI-labeled cells from the apical part of the pulp toward the injury site—cryostat sections. DiI-labeled cells in red color. Bright-field pictures **(M,P)**, dark-field pictures **(N,Q)**, merged pictures **(O,R)**. Black and white arrows indicate injured teeth. **(M–N)** Intact dental pulps 1 day after DiI injection (T1). Note that in DiI-positive cells remain in the apical part of the pulp. **(P–R)** Dental pulps 1day after cavity preparation and DiI injection (T1). Light blue arrowheads indicate the final location of migrating DiI-positive cells. Abbreviations: p, pulp; T0, time zero days; T1, time 1 day. Scale bars: 100 μm **(A,B,D–R)**, 20 μm **(C)**.

We then monitored the movement of dental pulp cells located at the apical part of the forming root in cultured first molars. For this purpose, DiI was injected in apical pulp cells (Figures 3A–C,G–I, 4A–C) of injured and intact molars (time of injection indicated as T0) and then the molars were cultured for up to 4 days (T4). The development of the dental explants proceeded normally during the culture period. After fixation of the dental explants, their hard tissues (i.e., dentin, enamel) were carefully removed in order to keep intact the morphology of the pulp tissue. The experiments were performed in molars from postnatal day 6–8 mouse pups since the integrity of pulp tissues from older mice was severely impaired upon removal of the dental hard tissues. Dental pulps were then photographed using a fluorescence microscope. After merging fluorescence and bright-field images, DiI-positive cells in injured teeth were observed not only in apical pulp cells but also in migrating cells that form a line joining the apical part with the injury site of the tooth (Figures 3D–F). In contrast, in pulps of intact teeth, labeled cells did not migrate and remained as cohesive patches in the apical pulp, where DiI was injected (Figures 3J–L). Similar results showing migration of DiI-positive cells from the apical part of the pulp toward the injury site were also obtained in tooth explants after 1 day of culture (Figure 4).

## Discussion

Severe dental injuries (i.e., deep cavity preparations) induce apoptosis of odontoblasts and activation of complex regenerative mechanisms within the dental pulp (Tziafas et al., 2000; Mitsiadis and Rahiotis, 2004; Mitsiadis et al., 2008, 2011). Cells of the sub-odontoblastic layer, which express the Notch2 receptor (Mitsiadis et al., 2003; Mitsiadis and Rahiotis, 2004), could replace the apoptotic odontoblasts and differentiate into odontoblast-like cells. However, sub-odontoblastic cells are equally eliminated by apoptosis after a severe stress. Therefore, the number of neighboring cells that are able to differentiate into odontoblast-like cells decreases considerably and alternative sources of pulp stem/progenitor cells should be activated to ensure dental tissue repair. Although dental pulp contains a significant amount of stem cells (Bluteau et al., 2008; Catón et al., 2011), severe injury might induce stemness in cells that in physiological conditions do not behave as stem cells (Potten and Loeffler, 1990). Human dental pulp stem cells (hDPSCs) isolated from permanent teeth (Gronthos et al., 2000) and the apical pulp of shed primary teeth (called SCAP) (Shi and Gronthos, 2003) are characterized by expression of several mesenchymal stem cell markers (e.g., CD29, CD73, CD105, CD44) and extracellular matrix molecules such as collagen, vimentin, laminin, and fibronectin (Bluteau et al., 2008; Pagella et al., 2015). hDPSCs are multipotent and have shown a great potential for repair and regeneration since they can differentiate into various cell types such as adipocytes (Waddington et al., 2009), chondroblasts/chondrocytes (Waddington et al., 2009), osteoblasts/osteocytes (de Mendonça Costa et al., 2008; Graziano et al., 2008), myocytes (Kerkis et al., 2008), cardiomyocytes (Gandia et al., 2008), and odontoblasts (Cordeiro et al., 2008; Nedel et al., 2009). This potential has made hDPSCs an attractive choice for tissue engineering, especially when they can be used as autologous transplants (Mitsiadis et al., 2015). The best example showing the success of such strategies is the first clinical trial using hDPSCs in patients for alveolar bone reconstruction (d'Aquino et al., 2009).

hDPSCs are thought to reside in one or more distinct storage sites, also called stem cell niches (Mitsiadis et al., 2015; Pagella et al., 2015). The concept of stem cell niches refers to defined anatomical compartments that include cellular and acellular (e.g., extracellular matrix) components. The niches provide highly specialized microenvironments, enabling stem cells to survive, self-renew, change their number and fate, and finally to participate in tissue repair (Scadden, 2006; Pagella et al., 2015). Similarly, niche-derived signals influence the function of dental pulp stem/progenitor cells. In addition, intercellular signaling pathways such as bone morphogenetic proteins (BMPs), Wnt, epidermal growth factor (EGF) and Notch are important regulators of stem cell function (Artavanis-Tsakonas and Muskavitch, 2010). Under the influence of these signaling pathways, dental stem cell might migrate from nearby or distant niches to the area of injury, where they will engraft and participate in the regeneration process. These cells will give rise to odontoblast-like cells that form the reparative dentin (Mitsiadis et al., 2015; Miran et al., 2016).

Significant molecular changes accompany dental pulp regeneration. For example, BMPs and transforming growth factor beta (TGFβ) are released from dentin after injury and contribute to reparative dentin formation (Tziafas et al., 2000; About and Mitsiadis, 2001; Mitsiadis and Rahiotis, 2004; Mitsiadis and Graf, 2009). Similarly, although Notch receptors are absent in adult dental pulps, their expression is re-activated after injury (Mitsiadis et al., 1999, 2003; Lovschall et al., 2007). In injured rodent teeth, the Notch1 and Notch3 receptors, as well as the Delta-1 ligand are expressed in cells related to vascular structures, either near or far away of the injury site, while Notch2 is strongly expressed in dental pulp mesenchymal cells at the root apex (Mitsiadis et al., 1999). A similar pattern of Notch2 expression was observed in injured human permanent teeth (Mitsiadis et al., 2003), suggesting the existence of a pool of putative stem/progenitor cells at the apical part of the pulp. Previous findings in injured teeth showed reactivation of Notch3 in pericytes that might be another source for dental pulp stem cells (Lovschall et al., 2007). This is in line with previous findings indicating the existence of putative dental pulp perivascular niches (Shi and Gronthos, 2003; Oh and Nör, 2015). However, it has not yet proven that these Notch expressing cell populations participate to the process of dentin/pulp regeneration and can differentiate into odontoblast-like cells after injury. The activation of Notch signaling in human dental pulp stem/progenitor cells *in vitro* by either Jagged1 or the intracellular domain of Notch1 leads to inhibition of odontoblast differentiation without affecting cell proliferation (Zhang et al., 2008). In contrast, the activation of Notch signaling by Delta1 stimulates both cell differentiation and proliferation (He et al., 2009). Together these results suggest that Notch receptors may act as both positive and negative regulators of DPSCs depending on the ligand that they bind. Proliferative events in the apical pulp correlate to Notch2 expression after tooth crown injury. Our results are in line with recent findings showing that Notch2 signaling controls proliferation of various stem cell populations (e.g., bone marrow stem cells) and cancer cells (Huang et al., 2015; Sato et al., 2016; Wu et al., 2016).

In intact teeth, no obvious movement of the DiI-labeled apical pulp cells was observed. In contrast, in injured teeth, these cells migrate from the apex toward to the wounded area, a site where the reparative dentin will form. The migrating cells were seen in the line unifying the apex with the injury site. These results indicate that the apical part of the pulp contains a pool of putative stem/progenitor cells capable of migrating toward the injured site of the tooth. Our findings show a correlation between DiI-labeled cells, proliferating cells and Notch2-positive cells at the apical part of the injured dental pulp, thus suggesting a regulatory role for Notch2 signaling in proliferation and migration of pulp stem/progenitor cells. Loss- and gain-of-function experiments will be needed to investigate the functional role of Notch2 signaling in this process.

**Figure 5:**
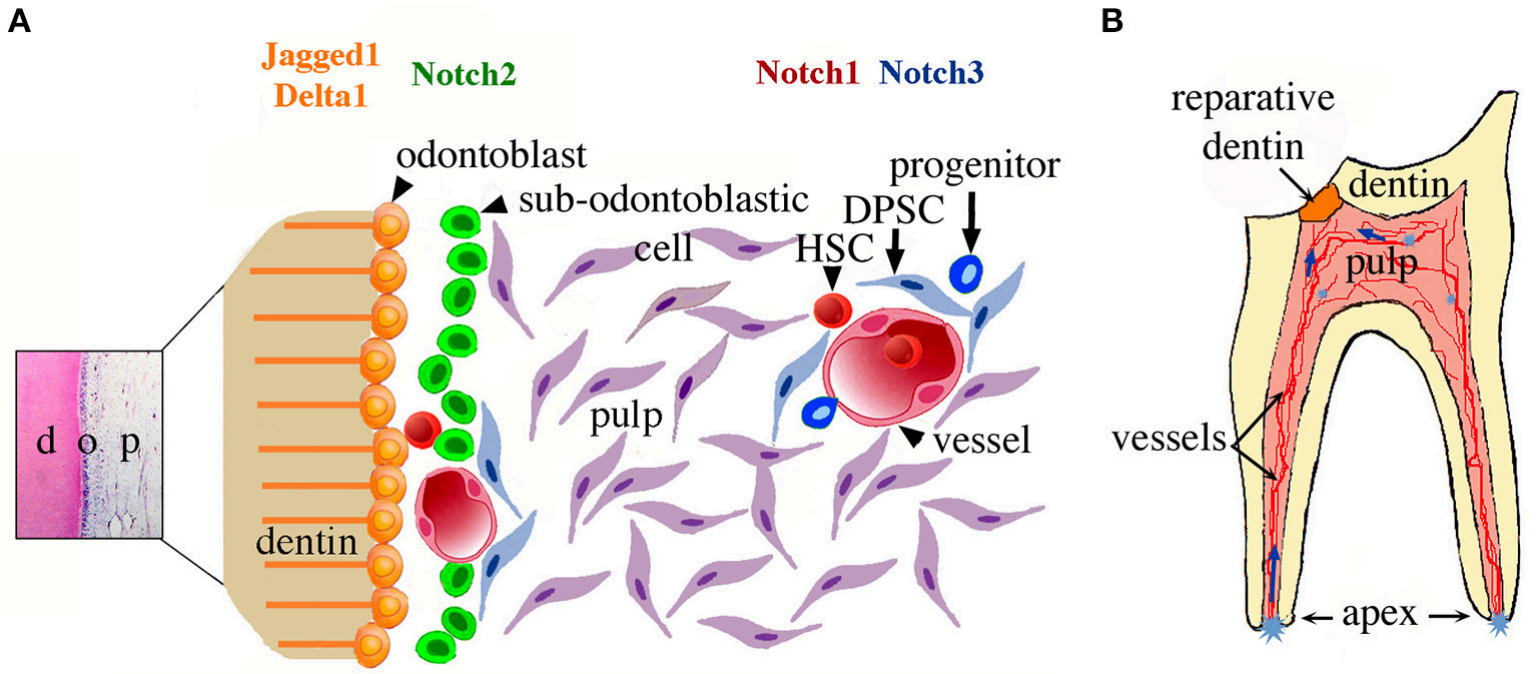
Schematic representation of the suggested relationship between Notch signaling, dental pulp stem cell niches, and their activation upon tooth injury. **(A)** Potential stem/progenitor cells within the dental pulp at the crown level. Cells related to perivascular niches and odontoblast progenitors (i.e., sub-odontoblastic cells) will express a specific Notch receptor after dental injury at the crown level. **(B)** A model summarizing the cellular events after dental injury. Cavity preparation activates stem cell niches located in different parts of the dental pulp (light blue asterisks). Stem/progenitor cells start to migrate toward the injury site (blue arrows) and once on place might differentiate into odontoblast-like cells and participate to the formation of reparative dentin. Abbreviations: d, dentin; DPSC, dental pulp stem cells; HSC, hematopoietic stem cells; o, odontoblasts; p, pulp.

Although cells located at the apical pulp can end up far away (i.e., tooth crown) and participate in healing, other pulp stem/progenitor cell populations may also participate in tooth repair (e.g., cells derived from the perivascular niches), either concomitantly with apical pulp cells or at different time points (Mitsiadis et al., 2011, 2015; Oh and Nör, 2015; Ducret et al., 2016). A hypothetical schematic model of the pulp stem cell niches, their activation upon tooth injury, and stem cell kinetics within the pulp during repair is shown in Figure 5. To determine whether Notch2-positive cells directly contribute to these cellular events upon tooth injury, genetic lineage tracing experiments using a Notch2-creERT driver (Fre et al., 2011), combined with immunostaining for odontoblast differentiation markers, have to be undertaken. The present findings highlight the importance and dynamic nature of the apical pulp cells for tooth repair. These cells can reach the injury site and may participate in the formation of reparative dentin. Here we establish a conceptual framework where important cellular and molecular mechanisms operating during dental regenerative processes start to be elucidated. Such knowledge might be helpful for the realization of innovative dental treatments.

## Author contributions

TM, Contributed to the conception of the hypothesis of the study and in the development of the model, involved in the evaluation of the results and preparation of the manuscript. He also provided approval for the publication of this version. JC, Contributed to the conception of the hypothesis of the study and to the development of the model, the acquisition and the analysis of data for the work. He was also involved in the preparation of the manuscript and provided approval for the publication of this version. PP, Contributed to the analysis and the interpretation of data for the work. He was also involved in the preparation of the manuscript and sanctioned the publication of this version. GO, Contributed to the development of the model and the interpretation of data for the work. She was also involved in the preparation of the manuscript and provided approval for the publication of this version. LJ, Contributed to the hypothesis of the study, collaborated in the development of the model and was involved in the evaluation of the results and preparation of the manuscript. She also provided approval for the publication of this version.

### Conflict of interest statement

The authors declare that the research was conducted in the absence of any commercial or financial relationships that could be construed as a potential conflict of interest.
